# Validating DNA Polymorphisms Using KASP Assay in Prairie Cordgrass (*Spartina pectinata* Link) Populations in the U.S.

**DOI:** 10.3389/fpls.2015.01271

**Published:** 2016-01-22

**Authors:** Hannah Graves, A. L. Rayburn, Jose L. Gonzalez-Hernandez, Gyoungju Nah, Do-Soon Kim, D. K. Lee

**Affiliations:** ^1^Department of Crop Science, University of Illinois at Urbana-ChampaignUrbana, IL, USA; ^2^Plant Science Department, South Dakota State UniversityBrookings, SD, USA; ^3^Department of Plant Science, Research Institute of Agriculture and Life Sciences, College of Agriculture and Life Sciences, Seoul National UniversitySeoul, Korea

**Keywords:** Prairie cordgrass, SNP, marker, *Spartina*, polymorphism, transcriptome

## Abstract

Single nucleotide polymorphisms (SNPs) are one of the most abundant DNA variants found in plant genomes and are highly efficient when comparing genome and transcriptome sequences. SNP marker analysis can be used to analyze genetic diversity, create genetic maps, and utilize marker-assisted selection breeding in many crop species. In order to utilize these technologies, one must first identify and validate putative SNPs. In this study, 121 putative SNPs, developed from a nuclear transcriptome of prairie cordgrass (*Spartina pectinata* Link), were analyzed using KASP technology in order to validate the SNPs. Fifty-nine SNPs were validated using a core collection of 38 natural populations and a phylogenetic tree was created with one main clade. Samples from the same population tended to cluster in the same location on the tree. Polymorphisms were identified within 52.6% of the populations, split evenly between the tetraploid and octoploid cytotypes. Twelve selected SNP markers were used to assess the fidelity of tetraploid crosses of prairie cordgrass and their resulting F_2_population. These markers were able to distinguish true crosses and selfs. This study provides insight into the genomic structure of prairie cordgrass, but further analysis must be done on other cytotypes to fully understand the structure of this species. This study validates putative SNPs and confirms the potential usefulness of SNP marker technology in future breeding programs of this species.

## Introduction

Prairie cordgrass (*Spartina pectinata* Link) is a native grass species of the North American Prairie that has a geographic distribution, ranging from the southern U.S. (Texas, Arkansas, and New Mexico) to northern Canada, and from the east coast through the Midwest to the western coast of the U.S. (Hitchcock, 1950; Voight and Mohlenbrock, 1979; Barkworth et al., 2007; Gedye et al., 2010). This species is adapted to a wide range of environmental conditions and, in addition, responds well to abiotic stresses, such as moderate salinity, water logged soils, drought, and cold tolerance (Montemayor et al., 2008; Boe et al., 2009; Gonzalez-Hernandez et al., 2009; Kim et al., 2011; Zilverberg et al., 2014; Anderson et al., 2015). Because of its wide adaptability, this warm season, C4, perennial grass is highly valued for conservation practices, wetland revegetation, streambank stabilization, wildlife habitat, forage production, and recently bioenergy feedstock production (Hitchcock, 1950; Barkworth et al., 2007; Montemayor et al., 2008; Gonzalez-Hernandez et al., 2009; Kim et al., 2011; Boe et al., 2013; Zilverberg et al., 2014; Guo et al., 2015). This ability to adapt to such a wide diversity of conditions results in populations becoming adapted to specific environments, ultimately leading to genetically diverse populations. Adding to the potential genetic diversity of prairie cordgrass is polyploidy.

Prairie cordgrass is a polyploid species, composed of three cytotypes: tetraploid (2*n* = 4*x* = 40), hexaploid (2*n* = 6*x* = 60), and octoploid (2*n* = 8*x* = 80) (Church, 1940; Kim et al., 2010, 2012). Because of the reproductive and geographic isolation between the cytotypes, there is likely an increase in polymorphisms and potential genetic diversity, especially within the tetraploid and octoploids cytotypes (Soltis et al., 1992; Wendel and Doyle, 2005; Hirakawa et al., 2014). There is a large amount of phenotypic variation present in all cytotypes of prairie cordgrass (Boe and Lee, 2007; Kim et al., 2012; Guo et al., 2015), but there is a lack of knowledge about the genomic structure. A few studies have revealed diversity within highly polymorphic chloroplast DNA regions observed within and among tetra- and octoploid populations (Kim et al., 2013; Graves et al., 2015). In prairie cordgrass, EST-SSR markers (Gedye et al., 2010), SSR (Gedye et al., 2012), and AFLP markers (Moncada et al., 2007) have been developed. However, these technologies may not be as cost-effective, scalable, successful, or as flexible as using single nucleotide polymorphisms (SNPs) (Semagn et al., 2014).

SNPs provide a highly efficient way to conveniently compare genomic and transcriptome sequences. Because they are one of the most abundant DNA variants found in plant genomes, SNPs are more likely to be related to specific biological functions and phenotypes (Rafalski, 2002; Bundock et al., 2006; Salem et al., 2012). This technology has been applied in genetic diversity analysis, genetic map construction, association map analysis, and marker-assisted selection breeding in many different types of crop species (Byers et al., 2012; Saxena et al., 2012; Semagn et al., 2014; Sindhu et al., 2014; Wei et al., 2014). SNP marker technology is also utilized in high-throughput genotyping, increasing the speed of the selection process by eliminating growing plants to maturity for phenotypic selection (Paux et al., 2012). In order to use SNP markers for genetic improvement, there is a three-step process one must follow: (1) SNP discovery after aligning sequence reads generated by next-generation sequencing technologies for different genotypes of a given species; (2) validate SNPs to distinguish DNA polymorphisms of actual allelic variants from those of other biological phenomena such as gene duplication events; (3) SNP genotyping of germplasm collection or genetic/breeding populations (Saxena et al., 2012).

Step one of the process was accomplished in prairie cordgrass by using a transcriptome assembly derived from multiple genotypes and tissues (Gonzalez et al., personal communication). The second and third steps are yet to be completed for polyploid prairie cordgrass. Several parameters, such as sample size, number of SNPs to be used for analysis, cost effectiveness, and the SNP genotyping platform, must be considered in these analyses (Semagn et al., 2014). Many technologies exist for use in SNP genotyping analysis, but one technology performs well when it comes to adaptability, efficiency, and cost-effectiveness. Kompetitive allele-specific PCR (KASP), developed by LGC Genomics (Teddington, UK; www.lgcgenomics.com), is a PCR-based homogeneous fluorescent SNP genotyping system, which determines the alleles at a specific locus within genomic DNA (Semagn et al., 2014). The KASP technology has been utilized on other polyploid plant species, including switchgrass (LGC Genomics, 2014), cotton (Byers et al., 2012), wheat (Paux et al., 2012), potato (Uitdewilligen et al., 2013), and various triploid citrus species (Cuenca et al., 2013).

In this study, SNPs, identified in the nuclear transcriptome, were converted to the KASP marker system in order to validate that these SNPs are true allelic variants. In addition, KASP markers were used in quality control analysis when making crosses, prairie cordgrass being a putative self-compatible species. The main objectives of this study were (1) to validate SNP polymorphisms identified in the nuclear transcriptome of natural populations of prairie cordgrass in the U.S. and (2) to assess the fidelity of specific tetraploid crosses and selfs, and to elucidate inheritance patterns of SNP markers.

## Materials and methods

### Development and validation of KASP genotyping assays

In a separate study by Gonzalez et al. (personal communication) at South Dakota State University, a transcriptome of prairie cordgrass was assembled using ~1.2 billion Illumina paired-end reads from various vegetative tissues (roots, leaves, and rhizomes) under various conditions (salt stress, cold stress, and differing photoperiods) in order to obtain an abundance in diversity, with regards to the number and type of transcripts. The assembly was developed using CLC Genomics Workbench 7.0 (Arhaus, Denmark) and annotated against the sorghum genes models. About 146,549 contigs, or transcript assemblies, of 230 bp or more with an N50 of 973 bp were used to mine over 1 million SNPs, insertions, and deletions using the variant detection function in CLC Genomics Workbench. Putative SNPs were filtered based on coverage (minimum of 100 X), a window of 80–100 bp free from additional SNPs and an allele frequency of 20–80%. Initially, nine bi-allelic SNPs were selected for analysis, associated with enzymes within the lignin biosynthesis pathway. Additional SNPs were selected without regard to putative function of the transcript assembly. A total of 121 bi-allelic SNPs were identified for use in this study (Table 1). SNPs were sent for primer development to be used in KASP genotyping assays. Genotyping with KASP was performed as follows.

**Table 1:** **Summary of SNP sequences, including SNP ID, SNP sequences, and SNP alleles**.

**SNP ID**	**SNP sequence/allele**	**SNP ID**	**SNP sequence**
pcg_00001	GTCCTTGAGCTCGGC**[G/A]**TCCACGTCCAAGCG	pcg_00032	GCCAGTATTGGCAAG**[A/C]**ATGCAACAATTACT
^*^pcg_00002	CGCCAGGTACACCGG**[C/G]**GCCGCCTGGTTAGT	pcg_00033	AAAGACTACCCTTCC**[A/C]**TATCGAATAGAGAA
pcg_00003	GTCGGCCCCGGCCTC**[A/G]**AACCACGGGACGCC	pcg_00034	ACAGCTCCGGATGAA**[A/G]**TGGTACTTGATCCG
pcg_00004	ACCCGAAGGAGAAGG**[G/T]**CGCGATGGCGCCCG	^*^pcg_00035	TCTTTCGACCAAGTA**[A/G]**CTCACCCAGTAGGC
pcg_00005	AAGAACAAATTTATA**[A/G]**GTTAAATACATGCA	^*^pcg_00036	GCTCGTGTCGATGTC**[G/A]**CCGGCGAGGTCGCT
pcg_00006	GCCAAAGGACAGATC**[A/G]**TGAATAACATGACT	pcg_00037	CGAGGTGTGATGCAC**[T/C]**AGAACGCCGCTCGT
pcg_00007	CGGAACTGAGGAACA**[A/G]**TAGCATACATGCTT	^*^pcg_00038	GCTCACATACCCGAC**[A/G]**GCGAACGCCAAGTC
pcg_00008	GTTCGACCGCGCGGC**[A/C]**ATCGCCGAGCTCGA	^*^pcg_00039	TGGGCAGGGTTGCAG**[T/C]**CACCCATGCCTCCC
pcg_00009	GAGAAGAAGAGAGTG**[A/G]**TTGCATCATTGGAC	^*^pcg_00040	CGCTTCTTCCGTGCC**[A/G]**GTGATGACGAGGTC
pcg_00010	GGTGCGGCTTGACAA**[T/C]**GTCACAATACAAGT	^*^pcg_00041	GTGTCCCCGGCCTCG**[C/T]**CGGTACACCGCCGC
pcg_00011	CTGTTTGTTAAGTGC**[A/G]**CTGAATTTGAGATT	pcg_00042	GCGGTGCTTGCCGCA**[A/G]**CCCGTACAAGGCCT
pcg_00012	GCATTCATGTTCCCA**[A/G]**TACATCCTGGCAAA	^*^pcg_00043	CTTCTTGAGCTTGAA**[T/C]**ACCCACTTCAGGGT
pcg_00013	ACAATCATTGTTTTT**[T/C]**GTAATTGGGGAACT	^*^pcg_00044	CGGGCGGTGGCCGGC**[T/C]**GGCAAGTCGACGAG
pcg_00014	CCAAATGGCAAAAAT**[T/G]**TACTCAGATTTCCA	pcg_00045	AAGTCAGTTGTTGTC**[T/A]**GCAACCCTCATCGT
pcg_00015	ACTTGATTTAGAGTC**[G/A]**GCAGACATCATTTT	^*^pcg_00046	TCTGTTTGATTACCA**[C/T]**GGTAAGCTCACTCA
pcg_00016	AGCGCTTCACGCGAT**[A/G]**GAGTTCTCCGAAAT	^*^pcg_00047	TTACCAAATACCCAG**[A/G]**TGCAGAGTTCAAGC
pcg_00017	TAGCTTTAGGTGTTG**[G/A]**GTTTCGCATCAGTA	pcg_00048	AAGCAACAACTTACT**[C/T]**GAGCAAAGTGCAAG
pcg_00018	AGAACCAACTCTTTA**[C/T]**ATCAGACTGCGTAT	pcg_00049	TTACTTTCATATAAC**[G/A]**GGATGAAGCATGCA
pcg_00019	AACAAAGACAACATG**[A/G]**CTCACGAGAAATTG	pcg_00050	CAGGGACATTCGTTT**[C/T]**GTCCTCCAAAAATA
pcg_00020	TTTGGATGTTGAACT**[G/A]**TCTCAGATGTCCTT	pcg_00051	CCCTTGAATGGCTTC**[T/C]**TTTTCTTTTGTGCA
pcg_00021	TTTGGATGTTGAACT**[G/A]**TCTCAGATGTCCTT	pcg_00052	CACCAACCACTTGTC**[A/G]**TGGTGACGCTTCGT
pcg_00022	ATGAATTTTGGCACG**[A/G]**ACTTTTTGTTTGAA	pcg_00053	CGAGGTTGATGTTTA**[T/C]**GCTCGTCGATGACG
pcg_00023	GCATCCACAAGAATG**[G/C]**CCATGAACAATTAA	pcg_00054	AAAGTATTTGTAGGA**[G/A]**ACCCCTGAGGGTTC
pcg_00024	GATCGAGAAAAAAAA**[A/T]**TTGGATGAAGATTC	pcg_00055	CTCGCGTGGCCTTCT**[C/G]**TGTCATAAACCATG
pcg_00025	TTTGAGGAGGACGGT**[G/A]**ATGATAGCAAATCT	^*^pcg_00056	GGAACGTATCCTGTG**[T/C]**ATAAGGGCTCTCCG
pcg_00026	GTGAGGGATAGATTG**[T/G]**CAAGCAATGCAAGT	pcg_00057	AACTTGGTATCAGAC**[C/G]**GCCAAGGTTAAACC
pcg_00027	CCATCTAAGGTCAGG**[A/G]**TTCTAAGTTCATTC	pcg_00058	GGCACGGTAAACCTT**[T/G]**GCAAAGGTCCCTTG
pcg_00028	ACATTCTTCCGATCT**[C/A]**GGGTTTTTAACCCA	pcg_00059	TCAACCGTCTCCCCC**[G/C]**AGATGATTGTCTAA
pcg_00029	GGACCATTTGTTGTC**[A/G]**TCAAGGTTTCCCAG	pcg_00060	CACCCCACAAGACCA**[T/A]**ATGTCGGCTTTTGC
pcg_00030	GAGAGCATTGATGTC**[G/A]**CTGGCTCTTGGAAA	pcg_00061	AAATCTTTTTTTCCA**[G/T]**TATCTTTTTTCTTA
pcg_00031	AGTTAGACCTGAGAT**[T/C]**GAACATTCTGAAAA	pcg_00062	GTAATTGTTTGCAGA**[C/G]**AACTTTTCATTTGT
pcg_00063	GGAAGATATGCAACA**[C/T]**TTTGGGGAGGAAGC	pcg_00093	TACTGGGAAGAAACC**[G/A]**TTCCACTTGTCCTG
pcg_00064	GGGGATGTCACCCTT**[C/T]**CCCGGCGCGGTGAT	pcg_00094	GCTCTCCGCACACGC**[C/T]**GCCACCGCTACATC
pcg_00065	CAGCGGCAGCGACGC**[G/A]**GCGCTCCTGAGCCC	^*^pcg_00095	TGGTGAAAAGGTCCT**[G/C]**ATCCAGTTTGAGGA
pcg_00066	CGGCTTCGACCCGCT**[C/G]**GGCCTGGCGGAGGA	^*^pcg_00096	CAGGGACCGGAACCG**[G/A]**TTCCACCGGTTCAG
pcg_00067	GAGGATGTTGTCGAG**[C/T]**TTGACGTCGCGGTG	^*^pcg_00097	TTTTGTTACAAAATA**[C/T]**GAGCAAGCTCTGTT
^*^pcg_00068	CAATCCTGGAAAGGA**[C/T]**CCACTAATGTTTGT	pcg_00098	GTACAATGTCTGGGC**[C/A]**AGTACTCCTAATGG
pcg_00069	TGAAGTAACTACTAA**[A/T]**ATAGTACTGTTGTA	pcg_00099	AAAAAAAAGATGATG**[A/T]**CAGGTTACAAATTG
pcg_00070	AGGCTCTCACGATCA**[T/G]**TCCGAGTCGCTGTC	^*^pcg_00100	GACTCTCTACGGCTC**[C/A]**TCCAGGCTCACCGC
pcg_00071	GGCAAGGCTTTTACA**[A/C]**AAGAAGTTGTCGAG	pcg_00101	AGTACATGCAGGAGG**[G/A]**GCATTCTCTTCCTT
^*^pcg_00072	GGAGTACAATGGAAA**[A/G]**CTTCATGTGCCTGG	pcg_00102	CCATTTGAATCTCAA**[G/A]**GCACTGACGTGAAC
pcg_00073	TTTCCCTGGATTTGG**[C/T]**CTGGGTCTTGTTAT	pcg_00103	GCTAGCTTTTGCGCC**[C/T]**CTATACATCTTTTC
^*^pcg_00074	CGAGCATATAATATG**[G/A]**CCCTAAAATGATGG	pcg_00104	CGTCCTCGTCGTCTT**[C/G]**TTCCTCTGGCTGCT
pcg_00075	CGGCCGCGAGGACTC**[G/C]**CCGCTCGACATCAT	pcg_00105	GCTTGTGCTCATGGA**[T/C]**GTGGTTCACAGCCA
pcg_00076	CATCCCCACCTACGT**[C/G]**GTCGGAGTCAATGC	pcg_00106	ATTGGTGCTGTTGCT**[G/C]**GACGTGAAGCTGAC
^*^pcg_00077	CTCCTGCACCACCAA**[C/T]**TGCCTCGCGCCCTT	pcg_00107	AGATGACGGAGTCGG**[C/A]**GACGACGTGGGAGC
pcg_00078	ATGGAGGGACACAGC**[C/A]**GGCAAAGTGGATGT	pcg_00108	CTCTTTGCGCATGTG**[G/A]**CTCTTTTCCAGGGC
pcg_00079	AGATTCTGATATTGA**[T/C]**TTGGATGACTATTC	pcg_00109	ACTCAGACCATTTTG**[A/G]**ACCACCTCAGATGT
pcg_00080	TGCGTATATTCTCCG**[T/G]**GGTGAGACCAAAAT	pcg_00110	TATGTTATCTCAATG**[T/G]**GATCTACACCTGCA
pcg_00081	GCTCGCCCTCGCAAC**[T/A]**ATCGGATCTTGCGC	pcg_00111	GCCGACGGGATGCGG**[C/G]**CGATTCACATTTGC
^*^pcg_00082	CTGGCTGTAGGAATG**[G/A]**CCTTTTCACCTGAA	pcg_00112	TGACCACATGCCATG**[A/G]**GTATCAAGCCTATT
pcg_00083	TGAAGTTATGTATGA**[T/C]**CTGAGAGCTAGTGG	pcg_1186	GACCTCGCAGAACAC**[T/C]**GCAGACATGACCTC
pcg_00084	AAGTTCGGGATCAGC**[A/T]**CCGTGTATTTGGGA	pcg_13880	TCAAGTACCTCACCG**[G/A]**CGAGGCCAAGGCTT
pcg_00085	CTTCTGAAGTCGGAA**[C/A]**TGCCATCAAACTGG	pcg_14142	CACGCAGTTGGGGGC**[C/G]**AGGATGAGGACGAC
^*^pcg_00086	AGGAGTATCCACCTG**[G/T]**AATAACACTTGTAC	pcg_2412	CACATTGCGATTAGC**[G/A]**TATCGATCATGAAA
^*^pcg_00087	CAACACAATGAATCG**[T/G]**ATTGGAAAAGGAAG	pcg_37652	ACTTGAAGAGAGACG**[C/A]**ATCTGAAGGCAGAT
pcg_00088	TTTACAAATGCATAA**[A/G]**ATCTATGTTGGTAA	^*^pcg_38909	CACGCAGTTGGGGGC**[C/G]**AGGATGAGGACGAC
pcg_00089	CGTACCTGCAGTTCA**[T/C]**GTTCGCCTACATCT	pcg_77221	GAGCTCGCCAGGCAC**[G/T]**CTGGCTTCTGTGGC
pcg_00090	GAGGGGTAGTAAGAA**[A/G]**ACAAAGGAGACGTG	pcg_7965	TGACCAGCCGCAGCA**[G/A]**CCGCTCGTGGTAGT
pcg_00091	GGTACATAGTTTGAT**[C/T]**CACCTCCCTTCCTC	pcg_80876	TGGCGTCGTAGGTGC**[G/A]**CCACGGAGGACGCG
^*^pcg_00092	ATGGGAAGACAGGTT**[T/C]**GCAGCTTCATTATT		

* *Failed primers*.

*Bold letters are actual SNPS (SNP alleles)*.

For all samples, each amplification reaction contained 50 ng template DNA, KASP V4.0 2x Master mix standard ROX (LCG Genomics, Beverly, MA, USA) and KASP-by-Design assay mix (LGC Genomics, Beverly, MA, USA). The PCR thermocycling conditions for all primers, except pcg_1186, was 15 min at 94°C followed by 10 cycles of 94°C for 20 s and 61°C for 1 min (dropping −0.6°C per cycle to achieve a 55°C the annealing temperature) followed by 26 cycles of 94°C for 20 s and 55°C for 1 min. The PCR thermocycling conditions for primer pcg_1186 was 15 min at 94°C followed by 10 cycles of 94°C for 20 s and 65°C for 1 min (dropping −0.8°C per cycle to achieve a 57°C annealing temperature) followed by 26 cycles of 94°C for 20 s and 57°C for 1 min. After amplification, PCR plates were read with a Spectramax M5 FRET capable plate reader (Molecular Devices, Sunnyvale, CA, USA) using the recommended excitation and emission values. Data was then analyzed using Klustercaller software (LGC Genomics. Beverly, MA, USA) to identify SNP genotypes.

### Core collection analysis

In order to validate SNP polymorphisms of prairie cordgrass using KASP, seeds and rhizomes of natural populations were collected from across the continental U.S.A. (Kim et al., 2013) and grown at the Energy Biosciences Institute (EBI) Farm, Urbana, Illinois, USA. Individuals from 38 of these populations were selected as core collection based on geographic distribution; and two plants from each population were sampled, for a total of 76 plants (Table 2). Leaf tissue samples were stored at −80°C until DNA extraction was performed. Total genomic DNA was extracted from frozen leaf tissue using the CTAB method (Mikkilineni, 1997) with slight modifications as described by Kim et al. (2013). Fifty-nine KASP genotyping assays out of 121 were selected and used to analyze the collection and five additional *Spartina* species samples, namely; *S. alterniflora, S. patens* (Flageo vt.), *S. patens* (Sharp vt.), *S. patens*, and *S. bakeri*. All of the KASP genotyping assay results were recorded as a two-letter code, or SNP code, i.e., AA, AG, GG. A DNA fingerprint was made using all the SNP genotypes creating a concatenated DNA-like sequence, which was then imported into MEGA 6 (Tamura et al., 2013) to make a phylogenetic tree. The maximum parsimony (MP) tree, inferred from 1000 replicates, was obtained using the Subtree-Pruning-Regrafting algorithm with a search level one in which the initial trees were obtained by the random addition of sequences (Felsenstein, 1985; Nei and Kumar, 2000). All positions with <95% site coverage were eliminated.

**Table 2:** **Summary of plant materials used including, location, cytotype, and number of plants used per population**.

**ID**	**Location**	**Ploidy**	**Number of samples**
103 4X	IL	4X	2
9046803	NY	4X	2
IL102	IL	4X	2
IL99A	IL	4X	2
MBB4X	IL	4X	2
PC09-101	CT	4X	2
PC09-102	CT	4X	2
PC17-109	IL	4X	2
PC17-111 4X	IL	4X	2
PC19-101	IA	4X	2
PC19-103	IA	4X	2
PC19-105	IA	4X	2
PC20-102	KS	4X	2
PC20-105	KS	4X	2
PC22-101	LA	4X	2
PC23-101	ME	4X	2
PC23-104	ME	4X	2
PC29-101	MO	4X	2
PC29-104	MO	4X	2
PC34-101	NJ	4X	2
PC40-101	OK	4X	2
PC55-102	WI	4X	2
PC55-103	WI	4X	2
ND-2-51-4	ND	8X	2
PC17_111 8X	IL	8X	2
PC19-106	IA	8X	2
PC19-107	IA	8X	2
PC19-108	IA	8X	2
PC20-104	KS	8X	2
PC20-106	KS	8X	2
PC27-103	MN	8X	2
PC31-101	NE	8X	2
PC31-104	NE	8X	2
PC38-101	ND	8X	2
PC40-104	OK	8X	2
PC46-110	SD	8X	2
PCG109	SD	8X	2
Red River	MN, SD, ND	8X	2
Total			76

### F_1_ cross

In order to assess the utility of the KASP marker system in confirming specific tetraploid crosses of prairie cordgrass, a reciprocal cross involving two individuals (PC17-109 × PC20-102) of two populations differing in morphological characteristics of potential agronomic importance was developed. PC17-109 is a tetraploid population from Illinois with a phalanx rhizome type and low seed mass, whereas PC20-102 is a tetraploid population from Kansas with a guerilla rhizome type and high seed mass. In a greenhouse, the female inflorescence was covered ~1 day prior to stigma emergence, while pollen was collected from the male parent. Pollen was directly applied to the stigmas with a brush, and rebagged until anthesis was completed. A total of 83 individuals, 70 F_1_ individuals from PC17-109 (female) × PC20-102 (male) and 13 F_1_ individuals from PC20-102 (female) × PC17-109 (male) were sampled. F_1_ seeds were planted in greenhouse setting. Leaf tissue samples of each seedling were collected and stored at −80°C until DNA extraction was performed. Total genomic DNA was extracted from frozen leaf tissue as described previously. For the F_1_ individuals, 12 KASP genotyping assays were selected based on the parental SNP genotypes (Table 3). All of the assay results were recorded as two-letter SNP codes. To determine if the F_1_ progeny followed segregation of a typical monohybrid cross in relation to SNP genotype, a χ^2^ analysis was performed using *P* = 0.05, *df* = 2, and χ^2^ critical value = 5.991. The observed, along with the expected genotype, was recorded for each KASP genotyping assay.

**Table 3:** **Primers selected for use on the prairie cordgrass F_**1**_ progeny**.

	**pcg_00011**	**pcg_00012**	**pcg_00024**	pcg_00050^*^	pcg_00058^*^	pcg_00059^*^	pcg_00106^*^	pcg_1186^*^	pcg_14142^*^	**pcg_00061**	**pcg_00062**	**pcg_7965**
PC17_109 (Parent)	GA	GA	TA	TT	GG	CC	CC	CC	GG	TG	GC	AG
PC20_102 (Parent)	GG	GG	AA	CC	TT	GG	GG	TT	CC	TG	GC	AG
13_F1001^†^	GG	GG	AA	CC	TT	GG	GG	TT	CC	TT	CC	AG
13_F1002	GA	GA	TA	TC	GT	CG	CG	CT	GC	GG	GG	GG
13_F1003	GA	GA	TA	TC	GT	CG	CG	CT	GC	GG	GG	AG
13_F1004	GA	GA	TA	TC	GT	CG	CG	CT	GC	TG	GC	AA
13_F1005	GA	GA	TA	TC	GT	CG	CG	CT	GC	TT	CC	AG
13_F1006	GA	GA	TA	TC	GT	CG	CG	CT	GC	TG	GC	AG
13_F1007^†^	GG	GG	AA	CC	TT	GG	GG	TT	CC	GG	GG	AG
13_F1008	GG	GG	AA	TC	GT	CG	CG	CT	GC	TG	GC	AA

*The first three primers indicate one parent as heterozygous and one parent as homozygous, the next six primers indicate both parents as homozygous for opposite alleles, and the last three primers indicate both parents as heterozygous. Also shown are SNP assay results for eight out of the 83 F_1_ hybrids. Indicated are samples that can be identified as true crosses and selfs*.

* *Primers that can distinguish true crosses from selfed samples*.

† *F_1_ individuals that are identified as selfs of the PC20_102 parent*.

### F_2_ self

To assess the utility of the KASP marker system in identifying selfed individuals in the tetraploid background and gauge the segregation pattern, F_2_ individuals were generated and genotyped. In a greenhouse, the prairie cordgrass inflorescence was covered ~1 day prior to stigma emergence with bags constructed to view progression of inflorescence development of F_1_ plants. When anthesis was reached, the bags were shaken to promote self-pollination. Bags remained until anthesis was complete. F_2_ seeds were collected and planted in a greenhouse setting. A total of eight F_1_ individuals were selfed (6 F_1_ of PC17-109 × PC20-102 and 2 F_1_ of PC20-102 × PC17-109) and 8–11 individuals were sampled from the planted seeds of each of the selfed plants (total of 76). Leaf tissue samples were stored at −80°C until DNA extraction was performed. All 12 of the KASP genotyping assays selected to score the F_1_ individuals were also tested on the F_2_ individuals. All of the assay results were recorded as a SNP code as done in the F_1_ analysis. All SNP codes that were not accurately identified were removed from analysis.

## Results

### Development and validation of KASP assays

Twenty-six (21.5%) SNPs failed KASP marker development. From the remaining 95 (78.5%), 11 SNPs were found to be monomorphic when tested on the core collection DNA, resulting in 84 SNPs that were true allelic variants. Three of the eleven monomorphic markers were selected to discover if future plant samples would reveal the SNP polymorphisms previously identified in the transcriptome. From the 84 allelic variants, 56 of the most highly polymorphic SNPs were selected for further use in this study, resulting in 59 total KASP genotyping assays (Table 4).

**Table 4:** **Sampling of DNA fingerprints created using SNP codes from 59 KASP genotyping assays**.

**Subject ID**	**pcg_00006**	**pcg_00008**	**pcg_00009**	**pcg_00010**	**pcg_00011**	**pcg_00012**	**pcg_00013**	**pcg_00014**	**pcg_00015**	**pcg_00016**	**pcg_00017**	**pcg_00018**	**pcg_00021**	**pcg_00022**	**pcg_00023**	**pcg_00024**	**pcg_00025**	**pcg_00026**	**pcg_00027**	**pcg_00028**	**pcg_00029**	**pcg_00032**	**pcg_00034**	**pcg_00037**	**pcg_00042**	**pcg_00049**	**pcg_00050**	**pcg_00058**	**pcg_00059**	**pcg_00060**
PC19_103_1	–	AA	AA	TT	GA	GA	CT	GT	AG	GA	AG	TC	AG	AA	CG	AA	GG	GT	GA	AC	GA	CA	GG	CC	GG	AG	TT	GG	CC	TT
PC19_103_2	–	AA	AA	TT	GA	GA	CT	GT	AG	GA	AG	TC	AG	AA	CG	AA	GG	GT	GA	AC	GA	CA	GG	CC	GG	AG	TT	GG	CC	TT
PC20_104_1	GA	–	–	–	–	–	CT	GT	AG	GA	AG	TC	AG	GA	CG	TA	AG	GT	GA	AC	GA	CA	GA	CT	GA	GG	CC	GT	GG	AT
PC20_104_2	GA	–	–	–	–	–	CT	GT	AG	GA	AG	TC	AG	GA	CG	TA	AG	GT	GA	AC	GA	CA	GA	CT	GA	GG	CC	GT	GG	AT
PC34_101_1	GA	CA	–	TT	GA	GA	CC	GG	AA	AA	AA	TT	AA	AA	CC	–	GG	GG	GG	AA	GG	CC	GG	CC	GG	AA	TT	GG	CG	TT
PC34_101_2	GA	AA	–	CT	–	GA	CT	GT	AG	–	AG	TC	AG	–	CG	TA	–	GT	–	AC	GA	CA	–	–	–	–	CC	GT	–	–
PC38_101_1	GA	CA	GA	–	AA	AA	TT	GT	GG	–	GG	CC	GG	GA	GG	TA	AG	TT	AA	CC	AA	AA	GA	CT	GA	GG	CC	GT	GG	–
PC38_101_2	GA	–	–	TT	GA	GA	–	GT	AG	–	AG	TC	AG	AA	CG	TA	GG	GT	GA	AC	GA	–	GG	CC	GG	AA	TT	GG	CC	AT
Red_River_1	AA	CA	GA	CT	GA	GA	CT	GT	AG	GA	AG	TC	AG	GA	CG	TA	AG	GT	GA	AC	GA	CA	GA	CT	GA	AG	CC	GT	GG	TT
Red_River_2	AA	AA	AA	CT	GA	GA	CT	GG	AG	AA	AG	TC	AG	AA	CG	AA	GG	GT	–	AC	GA	CA	GA	CT	GA	AG	CC	GT	CG	AT
PC17_109	GA	CA	–	TT	GA	GA	CC	GG	AA	AA	AA	TT	AA	AA	CC	TA	GG	GG	GG	AA	GG	CC	GG	CC	GG	AA	TT	GG	CC	TT
PC20_102	GA	AA	AA	TT	GG	GG	CC	GG	AA	AA	AA	TT	AA	AA	CC	AA	GG	GG	GG	AA	GG	CC	GG	CC	GG	AG	CC	TT	GG	TT
*S. alterniflora* (East)	AA	CC	AA	TT	AA	AA	CC	–	–	GG	GG	CC	AA	AA	CC	AA	GG	GG	GG	AA	GG	CC	GG	CC	–	GG	CC	GG	GG	AA
*S. bakeri*	AA	CC	AA	TT	AA	AA	CC	–	–	GG	GG	CC	AA	AA	CC	TA	GG	GG	GG	AA	GG	CC	GG	CC	–	GG	CC	GG	GG	AA
*S. patens* (Sharp)	AA	CC	AA	TT	AA	AA	CC	–	–	GG	GG	CC	AA	AA	CC	AA	GG	GG	GG	AA	GG	CC	GG	CC	–	GG	CC	GG	GG	AA
*S. patens* (Flageo)	AA	CC	AA	TT	AA	AA	CC	–	–	GG	GG	CC	AA	AA	CC	AA	GG	GG	GG	AA	GG	CC	GG	CC	–	GG	CC	GG	GG	AA
*S. patens*	AA	CC	AA	TT	AA	AA	CC	–	–	GG	GG	CC	AA	AA	CC	AA	GG	GG	GG	AA	GG	CC	GG	CC	–	GG	CC	GG	GG	AA
**Subject ID**	**pcg_00061**	**pcg_00062**	**pcg_00065**	**pcg_00066**	**pcg_00078**	**pcg_00081**	**pcg_00083**	**pcg_00084**	**pcg_00085**	**pcg_00088**	**pcg_00092**	**pcg_00093**	**pcg_00098**	**pcg_00101**	**pcg_00102**	**pcg_00103**	**pcg_00104**	**pcg_00106**	**pcg_00109**	**pcg_00110**	**pcg_00111**	**pcg_00112**	**pcg_7965**	**pcg_14142**	**pcg_1186**	**pcg_77221**	**pcg_13880**	**pcg_2412**	**pcg_37652**
PC19_103_1	GG	GG	GG	GG	AC	TT	CT	TA	–	AA	CC	GG	CC	GG	AG	TC	CC	CG	GG	GG	CC	AA	AG	GC	CT	GG	GG	GG	CC
PC19_103_2	GG	GG	GG	–	AC	TT	CT	TA	–	AA	CC	GG	CC	GG	AG	TC	CC	CG	GG	GG	CC	AA	AG	GC	CT	GG	GG	GG	CC
PC20_104_1	TG	GC	–	GC	AC	AT	TT	AA	AC	GA	CC	AG	–	GG	AG	CC	GC	–	GA	GT	CC	AA	GG	GC	CT	TT	GG	GG	CC
PC20_104_2	TG	GC	–	GC	AC	AT	TT	AA	AC	GA	CC	AG	–	GG	AG	CC	GC	–	GA	GT	CC	AA	–	GC	GT	TT	GG	GG	CC
PC34_101_1	TG	GC	AG	GG	AC	TT	CT	TA	AA	–	CT	GG	CC	GG	AA	TC	CC	CC	GG	GG	GC	AA	GG	GG	CT	GG	GG	GG	CC
PC34_101_2	TG	–	AG	GC	–	TT	CT	TA	CC	–	CT	GG	AC	AG	AG	CC	CC	CG	GG	GG	–	AA	–	–	TT	GG	GG	GG	CC
PC38_101_1	GG	GG	GG	GC	AC	TT	CT	TT	AC	GA	CT	AG	CC	AG	AG	TC	GC	CG	GA	–	CC	AA	GG	GC	TT	GG	GG	GG	CC
PC38_101_2	GG	GG	GG	GC	CC	TT	CC	TT	CC	GG	TT	GG	AA	–	AG	CC	CC	CC	GG	GG	GC	AA	AG	GG	CT	GG	GG	GG	CC
Red_River_1	TG	GC	AG	GC	AC	AT	CT	TA	CC	GA	CT	AG	AC	AG	GG	–	GC	–	GG	GT	GC	AA	GG	GC	CT	GG	GG	GG	CC
Red_River_2	TG	GC	–	GC	CC	AT	CT	TA	CC	GA	CT	AG	AC	GG	AG	CC	CC	–	GG	GT	GC	AA	AG	–	TT	GG	GG	GG	CC
PC17_109	TG	GC	AG	GG	CC	TT	CC	TT	AC	GG	TT	GG	AC	AG	AG	TC	CC	CC	GG	GG	GC	AA	AG	GG	CC	GG	GG	GG	CC
PC20_102	TG	GC	GG	GC	CC	AT	CT	TA	CC	GA	CT	AG	CC	GG	AA	CC	CC	GG	GA	GT	CC	AA	AG	CC	TT	GG	GG	GG	CC
*S. alterniflora* (East)	–	CC	GG	GC	CC	TT	TT	AA	CC	GG	TT	GG	AA	GG	GG	CC	–	CG	GG	TT	–	AA	GG	CC					
*S. bakeri*	TT	CC	GG	GC	CC	TT	TT	AA	CC	GG	CC	AG	AA	GG	GG	TT	CC	CG	GG	TT	CC	AA	GG	CC					
*S. patens* (Sharp)	TT	CC	GG	GC	CC	TT	TT	AA	CC	GG	CC	AG	AA	GG	GG	TC	CC	GG	GG	TT	CC	AA	GG	CC					
*S. patens* (Flageo)	TT	CC	GG	GC	CC	TT	TT	AA	CC	GG	CC	AG	AA	GG	GG	CC	CC	GG	GG	TT	CC	AA	GG	CC					
*S. patens*	TT	CC	GG	GC	CC	TT	TT	AA	CC	GG	CC	GG	AA	GG	GG	CC	CC	GG	GG	TT	CC	AA	GG	CC					

### Core collection

**Figure 1:**
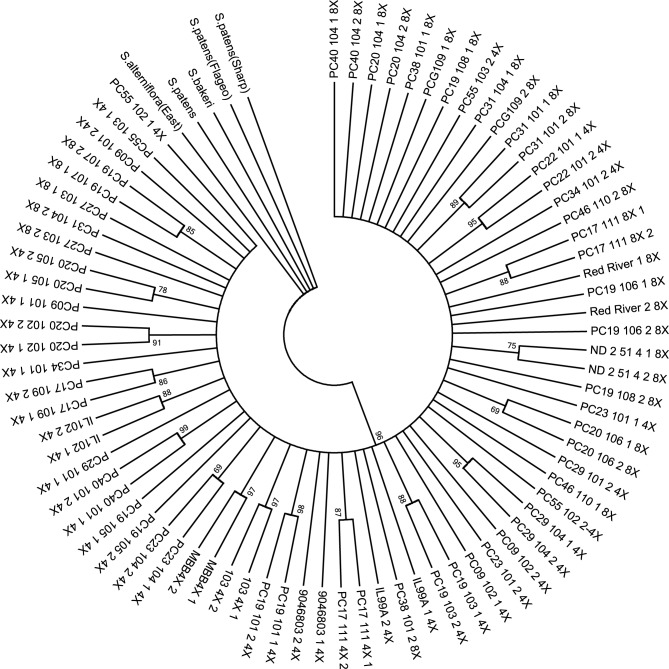
**Phylogenetic tree based on maximum parsimony analysis of combined SNP codes to create a DNA fingerprint sequence for the 38 prairie cordgrass core collection populations with a total of 76 plants and 5 ***Spartina*** outgroups**. Bootstrap values are indicated on the nodes as percentages. One main clade is identified.

The resulting data set from the DNA fingerprint contained 118 characters. There was an average of 3.8 missing character data points (SNP codes) per population. The maximum parsimony tree identified one clade after correcting for the missing data (Figure 1). For 47.4% of the populations, plants sampled from the same populations were observed to form subclades; however, intrapopulational variation was observed.

Out of the 38 prairie cordgrass populations, 52.6% showed polymorphisms within populations. Of the 52.6% polymorphic populations, 50% were octoploid and 50% were tetraploid. Out of the 15 octoploid populations sampled, 66.7% of the populations showed polymorphisms between the two plants sampled and 43.5% of the 23 tetraploid populations showed polymorphisms. The average number of polymorphisms that occurred within each population was 16. In the octoploid populations, 16.4 was the average number of polymorphisms observed, and 15.5 polymorphisms were observed as the average for tetraploids.

### F_1_ analysis

Only 6 out of 59 possible KASP genotyping assays showed both parents as homozygous SNPs but for opposite alleles. Three representative assays were selected which showed one SNP heterozygous for one parent and one SNP homozygous for the other parent, and three representative assays were selected which showed both parents as heterozygous SNPs (Table 3). All SNP codes that could not be accurately identified or called, due to not appearing in one of the three genotypes, were removed from the χ^2^ analysis. Four individuals did not consistently satisfy the expected heterozygous SNP genotype, with regards to KASP genotyping assays for which both parents were homozygous for opposite alleles (pcg_00050, pcg_00058, pcg_00059, pcg_000106, pcg_1186, and pcg_14142). These four individuals, after being analyzed across all 12 assays, were identified as being selfs, and were removed from the χ^2^ analysis (Table 3). Using the resulting trimmed data, the χ^2^ analysis indicated normal monohybrid 1:2:1 and 1:1 Mendelian inheritance patterns and could not be rejected for any of the primers (Table 5).

**Table 5:** **Summary of χ^**2**^ analysis on F_**1**_ progeny of a specific tetraploid prairie cordgrass cross with selfed data removed**.

**Primer ID**	**Observed X Allele**	**Expected X Allele**	**Observed Y Allele**	**Expected Y Allele**	**Observed XY Allele**	**Expected XY Allele**	**χ^2^**
**1:1 TEST**							
pcg_00011	0	0	44	39.5	35	39.5	1.025
pcg_00012	0	0	43	39	35	39	0.821
pcg_00024	43	39	0	0	35	39	0.821
pcg_00050	0	0	0	0	77	77	0
pcg_00058	0	0	0	0	77	77	0
pcg_00059	0	0	0	0	78	78	0
pcg_00106	0	0	0	0	75	75	0
pcg_1186	0	0	0	0	78	78	0
pcg_14142	0	0	0	0	79	79	0
**1:2:1 TEST**							
pcg_00061	27	19.8	15	19.8	37	39.5	3.962
pcg_00062	15	19.8	27	19.8	37	39.5	3.962
pcg_7965	17	19.8	20	19.8	42	39.5	0.544

*Analysis indicates that all primers produce expected results from a monohybrid Mendelian cross. df = 2, p = 0.05, critical χ^2^ = 5.991*.

### F_2_ analysis

The F_1_ parent genotype was identified in order to find SNPs that indicated the parent was homozygous (Table 6). For 3 F_1_ parents that were selfed, there were F_2_ progeny that did not fall into the expected homozygous parental genotype (example in Table 7). Two F_2_ progeny were identified consistently as unexpected offspring genotype of 13-F1008, 1 progeny of 14-F1014, and 4 progeny of 14-F1071. Individuals that consistently fell into the heterozygous (unexpected) genotype category across multiple homozygous primers were considered outcrosses and not true selfs of the F_1_(Table 7). Most of the F_2_ progeny were identified as expected SNP genotypes when considering the parental genotype.

**Table 6:** **SNP assay results for the F_**1**_ progeny used to determine SNP codes that could indicate true selfs in F_**2**_ progeny**.

**Parent**	**pcg_00011**	**pcg_00012**	**pcg_00024**	**pcg_00050**	**pcg_00058**	**pcg_00059**	**pcg_00106**	**pcg_1186**	**pcg_14142**	**pcg_00061**	**pcg_00062**	**pcg_7965**
13-F1008	GG^*^	GG^*^	AA^*^	TC	GT	CG	CG	CT	GC	TG	GC	AA^*^
13-F1011	GG^*^	GG^*^	AA^*^	TC	GT	CG	CG	CT	GC	GG^*^	GG^*^	AG
14-F1008	GA	GA	TA	TC	GT	CG	CG	CT	GC	TT^*^	CC^*^	AG
14-F1014	GG^*^	GG^*^	AA^*^	TC	GT	CG	CG	CT	GC	TG	GC	AG
14-F1015	GG^*^	GG^*^	AA^*^	TC	GT	CG	CG	CT	GC	TT^*^	CC^*^	AG
14-F1042	GG^*^	GG^*^	AA^*^	TC	GT	CG	CG	CT	GC	GG^*^	GG^*^	AG
14-F1067	GG^*^	GG^*^	AA^*^	TC	GT	CG	CG	CT	GC	TG	GC	GG^*^
14-F1071	GA	GA	TA	TC	GT	CG	CG	CT	GC	TG	GC	AA^*^

* *Indicates homozygous SNPs*.

**Table 7:** **SNP assay results for F_**2**_ individuals of two out of the eight selfed F_**1**_ samples**.

	pcg_00011^*^	pcg_00012^*^	pcg_00024^*^	**pcg_00050**	**pcg_00058**	**pcg_00059**	**pcg_00106**	**pcg_1186**	**pcg_14142**	**pcg_00061**	**pcg_00062**	pcg_7965^*^
13-F1008 (Parent)	GG	GG	AA	TC	GT	CG	CG	CT	GC	TG	GC	AA
F2:2012_13_F1_008_1	GG	GG	AA	TC	GT	CG	GG	TT	GG	GG	GG	AA
F2:2012_13_F1_008_2	GG	GG	AA	TT	GT	CG	GG	TT	GG	TG	GC	AA
F2:2012_13_F1_008_3	GG	GG	AA	TC	GT	CG	CG	CT	GC	TG	GC	AA
F2:2012_13_F1_008_4	GG	GG	AA	TC	GG	CC	CG	CT	GC	TG	GC	AA
F2:2012_13_F1_008_5^†^	GA	GA	TA	TT	TT	GG	CG	CC	GG	TT	CC	AG
F2:2012_13_F1_008_6^†^	GA	GA	TA	TC	GT	CG	CC	CC	GC	TG	CC	AG
F2:2012_13_F1_008_7	GG	GG	AA	TC	TT	GG	CG	CC	GC	TT	CC	AA
F2:2012_13_F1_008_8	GG	GG	AA	CC	TT	GG	CG	TT	GC	GG	GG	AA
F2:2012_13_F1_008_9	GG	GG	AA	TC	GG	CC	GG	CT	GC	TG	GC	AA
	pcg_00011^*^	pcg_00012^*^	pcg_00024^*^	**pcg_00050**	**pcg_00058**	**pcg_00059**	**pcg_00106**	**pcg_1186**	**pcg_14142**	pcg_00061^*^	pcg_00062^*^	**pcg_7965**
13-F1011 (Parent)	GG	GG	AA	TC	GT	CG	CG	CT	GC	GG	GG	AG
F2:2012_13_F1_011_1	GG	GG	AA	TC	TT	GG	GG	CT	GC	GG	GG	AG
F2:2012_13_F1_011_3	GG	GG	AA	TT	GT	CG	CG	CT	GC	GG	GG	AG
F2:2012_13_F1_011_4	GG	GG	AA	CC	GT	CG	GG	CT	GC	GG	GG	AG
F2:2012_13_F1_011_5	GG	GG	AA	TT	GT	CG	CG	CT	GC	GG	GG	GG
F2:2012_13_F1_011_6	GG	GG	AA	CC	GT	CG	CG	CC	GC	GG	GG	AA
F2:2012_13_F1_011_7	GG	GG	AA	CC	GG	CC	GG	CC	GC	GG	GG	AG
F2:2012_13_F1_011_8	GG	GG	AA	TC	GT	CG	CG	CT	GC	GG	GG	AA
F2:2012_13_F1_011_9	GG	GG	AA	TC	GT	CG	CG	CT	GC	GG	GG	AG
F2:2012_13_F1_011_10	GG	GG	AA	CC	TT	GG	CG	CC	GC	GG	GG	AG
F2:2012_13_F1_011_2	GG	GG	AA	TC	GT	CG	CG	CT	GC	GG	GG	AA

*Indicated are samples that can be identified as true selfs and as outcrossed*.

* *Primers that can distinguish true selfs from outcrossed samples*.

† *F_2_individuals that are identified as outcrossed samples*.

## Discussion

In order to validate SNP polymorphisms in prairie cordgrass, 121 SNPs identified from the nuclear transcriptome were sent for KASP assay development. Among 121 SNPs, the assay success rate was 78.5% with 26 assays failing development. This is comparable with findings in the literature of success rates of 83% (Cockram et al., 2012), 88.4% (Saxena et al., 2012), and 80.9% (Semagn et al., 2014). The assays failed mainly due to paralogs within the prairie cordgrass genome. Because not all of the populations used to develop the transcriptome were in the core collection of DNA used in this study, some assays appeared as monomorphic. These selected SNPs may have been derived from the octoploid populations not present in the core collection. Three monomorphic SNPs were selected for further analysis, to see if the SNPs would be polymorphic in future studies. With the failed and monomorphic assays removed, 84 putative SNPs were validated as true allelic variants and 59 SNPs were selected for this study. The 59 highly polymorphic assays were selected based on the criteria that there were at least two of the three genotypes present in a large portion of the samples analyzed. These assays were tested on the 38 natural populations, creating a phylogenetic tree that resulted in one clade containing all of the prairie cordgrass populations. If subclades were observed, the two plants of a single population were represented in the subclade.

Just over half of the populations showed polymorphisms within, with an equal number of octoploid and tetraploid populations. The average number of polymorphisms that occurred within each population did not vary between octoploid and tetraploid populations. This is different from a chloroplast DNA study of prairie cordgrass, in which there was little, if any, polymorphisms observed in the tetraploid cytotype (Graves et al., 2015).

SNPs were successfully identified in nuclear transcriptomes of prairie cordgrass and validated as allelic variants that can be used in prairie cordgrass. SNP markers were used to detect significant polymorphisms in prairie cordgrass populations collected from distinct geographic regions in the U.S. These SNP polymorphisms appear to reflect genetic relationships in prairie cordgrass and, therefore, can be used to assess genetic diversity within and among populations in future studies.

The F_1_ population, consisting of 83 plants, allows for the assessment of the fidelity of a specific tetraploid cross. Due to the lack of synchronization between the pollen and the ovaries, fewer seeds were obtained when PC20-12 was used as the female, compared with crosses involving PC17-109 as the female. Progeny that had SNP genotypes matching the female parent only were determined to be selfs. Of the F_1_ progeny, 95.2% were identified to be hybrids. Prairie cordgrass is a protogynous outcrossing species (Gedye et al., 2012), leading to the possibility that later-maturing stigmas could have been exposed to pollen from the same female parent, resulting in 4.8% of the F_1_ being selfs. The analysis of the 76 F_2_ progeny obtained by selfing eight F_1_ plants indicate that the SNPs, and the SNP markers chosen, could distinguish between a true selfed plant and an outcrossed plant. This is based on individuals consistently being genotyped as heterozygous (outcrossed) rather than being homozygous (selfed) as expected. Ninety-one percent of the F_2_ progeny were identified as successful selfs. Because of the protogynous nature of this species, there is already a natural element working against selfing. This could explain why outcrossed individuals were identified. There is also a possibility that some of the early-maturing stigmas were exposed to pollen in the greenhouse before bagging. This could explain why more F_2_ progeny were identified as unexpected genotypes (outcrosses) than the expected genotype (selfs) of the F_1_ progeny.

There is evidence that the tetraploid cytotype is an allotetraploid that may follow a disomic inheritance pattern. Two divergent copies in the *Waxy* lineages of *Spartina* genus support the allotetraploid origin of *S. pectinata* (Fortune et al., 2007). The bivalent pairing that occurs during meiosis (Church, 1940; Marchant, 1968a,b; Bishop, 2015) and the observation of disomic inheritance using genotyping-by-sequencing (Crawford, 2015) both suggest a disomic inheritance pattern in *S. pectinata*. This hypothesis was tested in a cross between two prairie cordgrass populations, exploiting the bi-allelic nature of the KASP technology to suggest Mendelian segregation ratios in a monohybrid type cross. The analysis of the F_1_ hybrids and F_2_ selfs conclude that disomic inheritance of SNPs in tetraploid prairie cordgrass is in agreement with the chromosomal and genomic evidence, and a possibility in this cross (Marchant, 1968a,b; Fortune et al., 2007; Bishop, 2015; Crawford, 2015).

The primary requirement of any breeding program is to ensure that accurate crosses are made (Glaszmann et al., 2010). The small flower size of prairie cordgrass and the large number of flowers per head make it hard to perform physical emasculation. Possibilities of self-pollination always exist and, therefore, developing a molecular way to confirm true crosses from selfs is warranted (Fang et al., 2004; Gedye et al., 2012). In prairie cordgrass, SSR markers have been developed that identified successful crosses in this protogynous species without the need for emasculation. This study also confirms that hybrids of prairie cordgrass can be created and verified with molecular markers. However, utilizing SSRs can be time-consuming, limited in number, and more expensive than SNP markers, making a way for the introduction of these newly developed and validated KASP assays.

## Conclusion

This study reports the first research of SNP marker development for use in prairie cordgrass. SNP markers developed from the nuclear transcriptome were tested on a core collection of DNA and found to be polymorphic among and within populations. The amount of variation differs from previous findings based on chloroplast DNA, which identified the octoploid cytotype as the most variable. However, one must recognize these SNP markers cover a wide range of expressed genomic DNA vs. two non-coding chloroplast DNA regions, giving nucleic SNP markers an advantage in identifying random genetic variation. These markers were used to assess the validity of true crosses that were made between two different populations using F1 and F2 (selfs of F1) progeny. Utilizing the biallelic nature of the KASP system, χ^2^ analysis of the F_1_ samples suggests that tetraploid prairie cordgrass may follow Mendelian disomic inheritance although other modes of inheritance were not ruled out. This analysis provides insight into the genomic structure of this species, supporting the hypothesis that tetraploid prairie cordgrass is an allotetraploid. However, further analysis must be done on other cytotypes to completely understand the genome structure of this species and to evaluate genetic diversity. In addition, this study underlines the usefulness of using SNP marker technology in future breeding programs of prairie cordgrass, and opens up the ability for the final step using SNP markers in genotyping germplasm collections or genetic/breeding populations of prairie cordgrass.

### Conflict of interest statement

The authors declare that the research was conducted in the absence of any commercial or financial relationships that could be construed as a potential conflict of interest.
